# Global health initiatives in Africa – governance, priorities, harmonisation and alignment

**DOI:** 10.1186/s12913-016-1448-9

**Published:** 2016-07-18

**Authors:** Aziza Mwisongo, Juliet Nabyonga-Orem

**Affiliations:** Health Systems and Services Cluster, World Health Organization Regional Office for Africa, B.P. 06, Brazzaville, Congo

**Keywords:** Global health initiatives, GFATM, GAVI Alliance, MAP, PEPFAR, Africa

## Abstract

**Background:**

The advent of global health initiatives (GHIs) has changed the landscape and architecture of health financing in low and middle income countries, particularly in Africa. Over the last decade, the African Region has realised improvements in health outcomes as a result of interventions implemented by both governments and development partners. However, alignment and harmonisation of partnerships and GHIs are still difficult in the African countries with inadequate capacity for their effective coordination.

**Method:**

Both published and grey literature was reviewed to understand the governance, priorities, harmonisation and alignment of GHIs in the African Region; to synthesise the knowledge and highlight the persistent challenges; and to identify gaps for future research.

**Results:**

GHI governance structures are often separate from those of the countries in which they operate. Their divergent funding channels and modalities may have contributed to the failure of governments to track their resources. There is also evidence that basically, earmarking and donor conditions drive funding allocations regardless of countries’ priorities. Although studies cite the lack of harmonisation of GHI priorities with national strategies, evidence shows improvements in that area over time. GHIs have used several strategies and mechanisms to involve the private sector. These have widened the pool of health service policy-makers and providers to include groups such as civil society organisations (CSOs), with both positive and negative implications. GHI strategies such as co-financing by countries as a condition for support have been positive in achieving sustainability of interventions.

**Conclusions:**

GHI approaches have not changed substantially over the years but there has been evolution in terms of donor funding and conditions. GHIs still largely operate in a vertical manner, bypassing country systems; they compete for the limited human resources; they influence country policies; and they are not always harmonised with other donors. To maximise returns on GHI support, there is need to ensure that their approaches are more comprehensive as opposed to being selective; to improve GHI country level governance and alignment with countries’ changing epidemiologic profiles; and to strengthen their involvement of CSOs.

## Background

The advent of global health initiatives (GHIs) has changed the landscape and architecture of health financing in low and middle income countries, particularly in Africa [1]. GHIs arose as a funding mechanism out of the need to advocate, mobilise and hasten funding, for some key health problems facing the globe [2]. More than 100 GHIs have been created over the last 20 years with the aim of assisting countries to achieve their health outcomes [2]. GHIs have mainly targeted disease conditions that affect poor countries, saving many lives [2].

In the last decade the African Region has seen improvements in health outcomes resulting from the substantial efforts of both governments and development partners [1, 3–6]. Besides multilateral and bilateral assistance, which are important sources of funding for health development, the African Region continues to register an increasing number of health partnerships and initiatives. This has made the aid architecture complex [7, 8].

Although partnerships and health initiatives provide an opportunity for health sector development, the variety of their funding levels, instruments for engagement with countries, focus, and scope of support creates challenges for the recipient countries [9–16]. The large number of health partnerships and initiatives also generates a wide range of issues and concerns in ensuring that they are aligned to sector priorities, and in preventing overburdening of government officials with extra demands [17–23]. GHIs are renowned for their large funding to countries. For example, three prominent GHIs, the World Bank’s Multi-country HIV/AIDS Program (MAP), the Global Fund to Fight AIDS, Tuberculosis and Malaria (GFATM) and the US President’s Emergency Plan for AIDS Relief (PEPFAR) have provided more than two-thirds of all direct external funding for scaling up HIV/AIDS prevention, treatment and care in resource-poor countries [15].

The term global health initiative has been a subject of debate [24]. Nervi [25] claims that several initiatives that have identified themselves as global are in fact bilateral and involve only one recipient country. There are contentions regarding GHI management approaches, particularly in regard to the fact that though several GHIs have their distinct governance structures, they require the services of the same human resources that support the health sector in the countries, which is viewed as burdensome [20, 26]. There have also been concerns about the nature of support rendered by GHIs, particularly regarding its focus, level, scheduling and timing, conditions and restrictions, all of which are thought to water down the positive synergism of GHIs [13, 22].

Alignment and harmonisation of partnerships and global health initiatives with national priorities are still challenges for the African countries with inadequate capacity for effective coordination of such undertakings [20, 21, 27]. In their stewardship efforts to strengthen health systems, governments are sometimes overwhelmed by partnerships and initiatives that have parallel approaches, which cause fragmentation of resources and hamper the holistic implementation of national health strategic plans [13, 21, 22, 28]. Partnerships and their members have peculiar priorities and ways of working and, consequently, consensus is not always achieved at the country level [28]. The partners’ unique reporting frameworks, funding cycles, focus and scope make aligning interventions for health systems strengthening difficult [15, 29, 30]. In addition, there are concerns that an increasing number of initiatives focus on issues, themes or diseases rather than on comprehensive approaches to health development and health systems strengthening [13, 22, 26, 31]. In this regard, there is a need to foster coherence of partnerships and health initiatives in the African Region in order to improve their complementarity and alignment. In addition, it is important to streamline partners’ efforts for harmonised action and greater mutual accountability and, to minimise duplications [22]. With Africa now embarking on the Sustainable Development Goals (SDGs) and aiming for universal health coverage, better understanding of GHI governance, priorities, harmonisation, and alignment is crucial. This will help develop strategies to improve GHI usefulness and impact on health. This paper mainly aims to help familiarise African governments with the peculiarities of the governance, priorities, harmonisation and alignment of GHIs for better implementation of their activities.

## Methods

This was a rapid literature review. A formal definition or methodology does not exist for a rapid literature review [32], but authors such as Khangura et al. [33] believe that it is becoming important as a tool to inform policy-makers and decision-makers on specific topics. The term “evidence summaries” is preferred to literature review. Characteristically, a rapid review is a short overview of available literature for a research question or set of research questions related to a single topic. For this study, the operational definition of GHIs was based on their possession of the following characteristics:

- Addresses a major health issue of international concern
- Is an organised effort linking people, partners and organisations
- Targets several countries
- Focuses on specific diseases or selected interventions, commodities or services
- Has the ability to generate resources for a country
- Is time limited

The main objectives of this review were to (1) explore and understand GHI governance processes, priorities, and harmonisation and alignment with country priorities in the African Region, (2) summarise and document these findings, and (3) identify gaps in the literature for future research and systematic reviews. The first two objectives were relevant for improved functioning of GHIs in Africa.

Two researchers conducted the rapid review. They first agreed on the research question, including the typology, and then on the databases to search and the search terms. They searched all the relevant literature related to GHIs and/or global health partnership (GHP) and low income countries (LMICs). The databases used were PubMed, Web of Knowledge, Web of Science, Google Scholar as well as websites of English language publications. Both peer-reviewed and grey literature was included in the search, with preference and emphasis given to literature focusing on the implementation and operations of GHIs in developing countries and Africa. Twenty three papers were initially identified, thereafter the researchers made use of their reference lists to obtain an additional 27 papers. All types of studies in Africa on the subject of interest were included in the review. Both researchers extracted the data from the literature and collated and summarised them according to the themes of interest.

## Results

### GHI governance structures

GHIs use various aid modalities in the countries they support and are generally criticised for their vertical governance structures [29]. They have a tendency to create their own country-level coordination groups or committees and programmes [34]. The four main GHIs, the GAVI Alliance, GFTAM, MAP and PEPFAR, for example, all have their distinct governance structures at the country level. The GAVI Alliance has a secretariat and a board at the global level but no staff in the countries. Decisions on vaccines are usually made by the Inter-agency Coordination Committee formed specifically for GAVI Alliance. In the case of the Global Fund, a secretariat and a board oversee GFATM’s functioning while a country coordinating mechanism and local fund agents operate at the country level [35]. United States’ GHIs have global AIDS coordinators and country teams coordinated through United States embassies. MAP is coordinated by the Global HIV/AIDS Programme and regional teams such as the AIDS Campaign Team for Africa and the South Asia Regional AIDS Team; the World Bank country director and national governments.

The persistent use of separate structures by GHIs is prompted by the weak state of the governance systems in Africa and other developing countries [1], the need to ensure effective use of funds, wider involvement of stakeholders, and to affect performance and outcomes. Evidence, however, shows that the separate and different structures have several negative consequences [8, 13, 29], including that (1) the countries are overburdened with the parallel and duplicative processes from the many GHIs [1]; (2) GHIs often bypass the existing donor coordination processes in the countries such as the Sector-wide Approach (SWAp) and the general budget support (GBS) mechanisms [29]; and (3) GHI operations generate the risk of competition for the limited skilled workforce [29, 36]. It is clear that GHIs are preferred to governments as employers owing to their lucrative enumeration and incentives [13, 36].

From the literature, GHIs are viewed as operating in a top-down manner with many of their restrictions being imposed from the global level. A vast majority of them are governed by boards consisting of a variety of partners with different backgrounds, aims and perspectives [25].

One of the main thrusts of GHIs is to foster involvement of civil society organisations (CSOs) in their activities, and they have several modalities for this [15]. Two common strategies are through CSO membership in GHI national committees and as recipients of GHI funds. Despite their good intentions, these approaches have been viewed as challenging, owing to the difficulties in ensuring CSO representativeness, capacity and accountability [13].

### Funding modalities

**Fig. 1:**
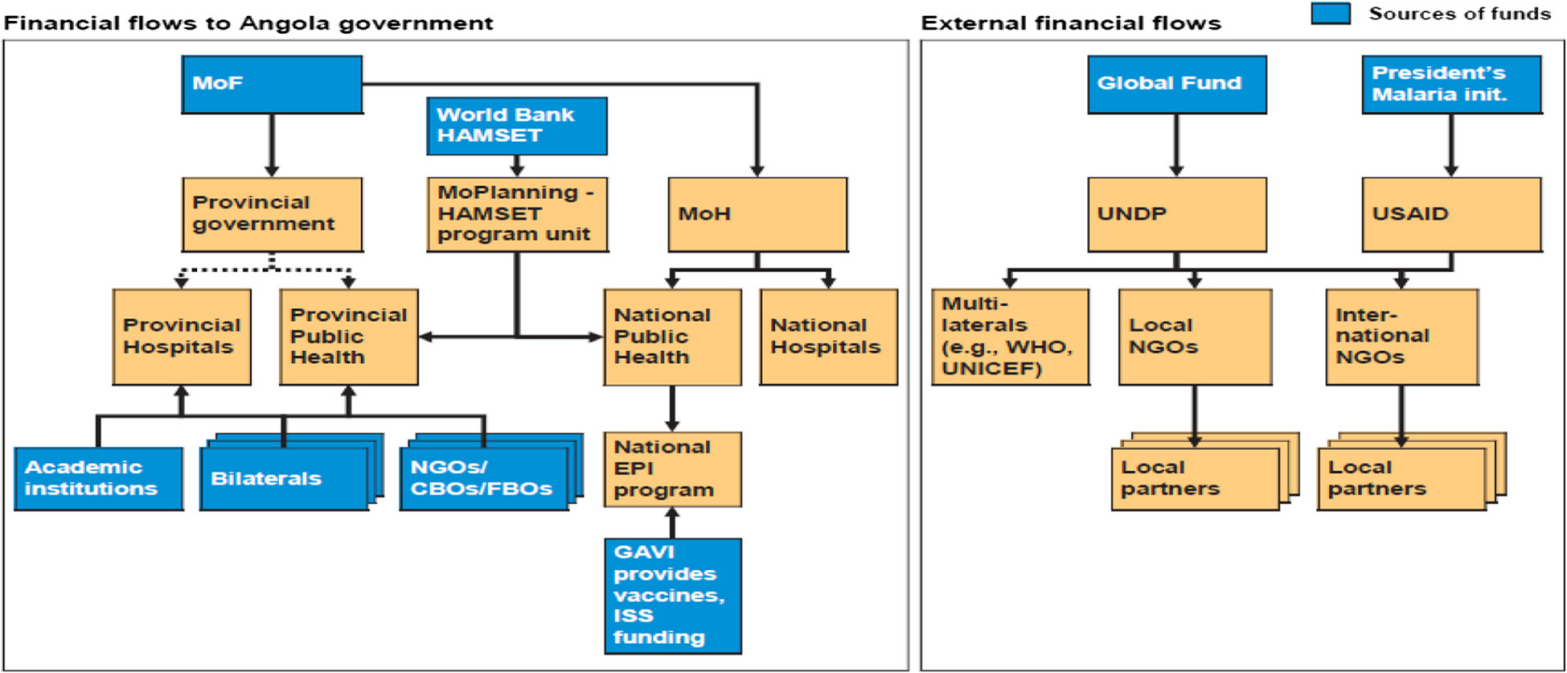
Fragmented financial flows for MOF, Global Fund and PMI in Angola. Source: Global Health Partnerships, Assessing Country Consequences, 2005

Most GHI funding support is earmarked for specific areas, with HIV being more commonly supported. Earmarked support is off budget support with multiple reporting systems. GHIs are concerned about good governance, and in their quest to ensure that funds are allocated and reach the intended beneficiaries, they have created many fragmented channels [29]. For example, in Angola the resource flows for funds from GFTAM and the President’s Malaria Initiative are completely distinct from those of the Ministry of Finance [37]. This poses difficulties in tracking resource flows, as illustrated in Fig. 1.

Examples from Burkina Faso and Mozambique illustrate how GHIs circumvent local financial systems in favour of their discrete systems. In Burkina Faso, the GAVI Alliance and GFTAM used a demand-driven approach to funding rather than the government’s SWAp system, which integrates single reports for all donors and provides decentralised funding to districts [38]. This was also observed in Mozambique, where the GAVI Alliance and PEPFAR support was not handled through the Ministry of Finance’s management process but by representatives or officials designated by the Ministry of Health [38]. However, there have been efforts by GHIs to reduce transaction costs, for example by providing resources commensurate with the challenge of absorbing aid, complementing existing country processes or systems, and improving communication.

### Areas of support

GHIs mainly focus on communicable diseases [13, 34, 39]. A study by the World Health Organization Maximizing Positive Synergies Collaborative Group found out that HIV/AIDS had disproportionately the greatest GHI support compared with other diseases [13]. In fact, 60 % of the GHIs in that study targeted the big three diseases of HIV/AIDS, tuberculosis and malaria, with HIV/AIDS attracting the most GHIs [34, 36]. It is reasonable to justify the concentration on HIV/AIDS, since it was responsible for high levels of mortality and morbidity before the introduction of antiretroviral drugs. By 2007, GFTAM, PEPFAR and MAP were contributing more than two-thirds of all external funding for HIV/AIDS and malaria in low resource countries [40]. This funding was committed to several areas that target HIV and related conditions such as tuberculosis [4]. Table 1 summarises the main characteristics and HIV/AIDS commitments of the three GHIs.

**Table 1:** Main characteristics and HIV/AIDS commitments of three GHIs, 2000–2004

	World Bank	Global Fund	PEPFAR
GHI type	Multilateral agency	Public-private partnership	Bilateral donor
Start	2000 (fiscal year 2001)	2002	2003 (fiscal year 2004)
Focus disease	HIV/AIDS	HIV/AIDS, tuberculosis and malaria	HIV/AIDS
Priority	Use of national AIDS strategic plans for setting priorities	Flexible funding based on priorities set by country stakeholders	Achieving programmatic targets set by the US Congress
Management system	National AIDS council (NAC) and NAC secretariat	Global Fund secretariat country	Office of the US Global AIDS Coordinator (OGAC)
Funding allocation	Based on government and NAC	Performance-based funding	Predetermined earmarked funding
Types of funded interventions	Community responses and capacity building	Proportion to intervention	Proportionate to treatment and prevention
Principle recipients	Multisectoral, government ministries, NAC, civil society	Government, NAC, civil society	International NGOs
Disbursement funding HIV/AIDS (millions of USD			
2003	307.7	789.1	949.2
2006	36.1	1031.3	2517.6

Sources: OECD CRS database, Oomman et al. 2007

GHIs are also renowned for the part they play in raising the profile of most neglected diseases such as onchocerciasis, dengue, trachoma, tetanus and schistosomiasis. Evidence shows that GHIs have programmes for these diseases, which in the past have had a low political profile and prioritisation [41]. GHIs have three broad strategies for supporting neglected diseases: raising the profile of the diseases, improving the delivery of interventions, and donating drugs. But support for neglected diseases in developing countries is viewed as unsustainable, and the consequences can be dire where support programmes are terminated. For example, Uganda faced major problems with drug shortages for neglected diseases after the drug donation period ended [34].

GHIs do not address all the gaps in health provision. For example, very few GHIs focus on non-communicable diseases [13]. There have also been concerns that most GHIs do not deal with certain areas that have a significant contribution to disability-adjusted life year (DALYs) losses such as maternal and reproductive health, depressive disorders, alcohol dependence and road traffic accidents, or the rising cancer incidences [13]. Some of the literature argues that although technical support to countries is crucial, GHI support has not been very successful [29]. Some reasons for this include the unclear and lack of a structured approach to identifying the gaps, and coordinating demand and supply to the identified technical assistance needs. Technical assistance has been ad-hoc and driven by urgent and immediate needs at the country level, an approach that often is unsustainable [42]. According to Biesma et al. [18], there were also unmet needs in the technical support provided by GHIs, such as in linking planning and disease prevalence, training on management and planning, cost-benefit analyses, monitoring and evaluation, and across-country application of lessons. GHIs have been blamed for selectively supporting certain groups that are easy to reach, contributing to the widening of the inequity gap [12, 29]. Much of the literature shows that GHIs are not completely aligned with the countries’ national strategic plans and often impose preconceived ideas [12, 29, 37].

### Funding levels

Despite the structural adjustment programmes and the worldwide recession, developing countries have seen a rise in development assistance for health since the early 2000s [3]. From studies mapping GHIs and GHPs, it is evident that Africa has the most GHPs per country followed by Asia. Eastern and central Europe have the lowest numbers of GHPs [28]. There are indications that a correlation exists between the number of GHPs operating in a country and its per capita GDP [34]. In general, the lower the per capita GDP, the greater the number of GHPs in the country, although there are inconsistencies [34]. The development assistance for health per DALY for HIV/AIDS, tuberculosis and malaria has increased in particular. Between 2002 and 2006, 32 % of the official development assistance for health was for HIV/AIDS and mostly went through the main GHIs for HIV/AIDS, tuberculosis, malaria and childhood immunisation, including polio. In 2007, investment through these GHIs accounted for two-thirds of all external funding for HIV/AIDS, 57 % for tuberculosis and 60 % for malaria [13].

**Fig 2:**
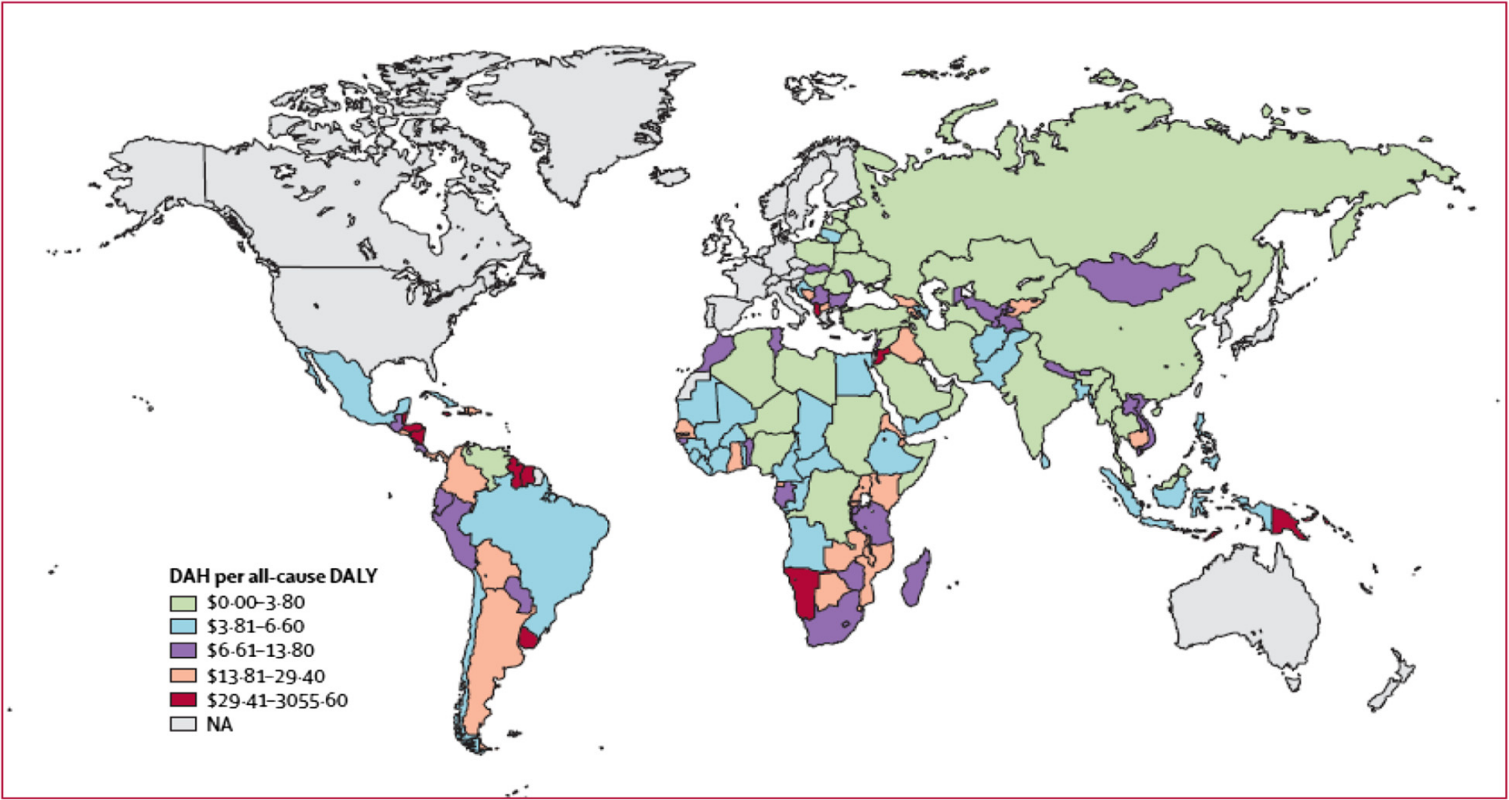
Map showing cumulative 2002–07 development assistance for health (DAH) per all-cause disability-adjusted life-year (DALY). Source: Ravishankar N. et al. Financing for global health: tracking developmental assistance for health from 1990 to 2007: Lancet 2009; 373: 2113-24

Substantial differences exist in the countries that have received GHI funding [43]. There is considerable variation in development assistance for health per DALY across regions and within regions as shown in Fig. 2. This variability is highly influenced by income, burden of disease, political stability, and historical and political relations between specific donors and recipient countries [43].

GHIs include NGOs and CSOs in their support. Although the channelling of funds through NGOs by GHIs has been riddled with controversy, a substantial portion of GHI funding is earmarked for NGOs and CSOs [44]. For example, GFTAM had allocated 30 % of all its grants to civil society groups in the countries of its support [44]. GHIs contribute directly to raising the overall health budgets of countries through their funding, but in a few cases they have done so through other processes [18]. For example, in Benin GFTAM contributions raised the overall budget for health spending by about 15 % [18].

The conditions relating to funding disbursement and performance have put immeasurable pressure on some weak economies [22, 29, 45]. In some cases the countries have failed to withdraw the funds allocated to them by GHIs owing to the strictness of the associated conditions, such as the requirement for performance-based reporting and to follow strict quality assurance guidelines [46]. Tanzania is a good example of a country where the conditions imposed by the GFTAM and MAP posed challenges in accessing their funds [46].

At the implementation level, delays in disbursement of funds and bureaucratic processes affect the use and absorptive capacity of funds, sometimes necessitating their return to the GHIs [46]. There are concerns also that in future GHI support might have adverse implications on sustainability and macroeconomic stability at the country level owing to the selective nature funding [47, 48]. This is in reference to activities or programmes initiated by GHIs that the countries are eventually forced to sustain, which might distort their funding architecture. Recently, however, some GHIs have introduced co-financing as a means to influence governments to contribute to activities, in a way ensuring some form of sustainability [31]. For example, the GAVI Alliance requires that the countries contribute to the procurement of some of their required vaccine doses. This policy encourages them to plan for financially sustainable immunisation programmes once GAVI Alliance support for new vaccines is phased out [31].

### Alignment of GHIs with national strategic plans and priorities

There has been growing concern in various African countries over the alignment of GHI objectives with those of the national strategic plans [2, 17, 19, 21]. One school of thought is that by nature, GHIs with their specific earmarked funding, inevitably will influence the countries that are highly donor dependent. GHIs’ disease focus has resulted in their shift from general health systems support [14, 21, 37, 49].

Examples exist on how GHIs have imposed restrictions on countries, such as the rejection of Ugandan’s 2002 Round One crosscutting, systems-strengthening proposal by GFTAM in favour of a more disease-specific proposal [16]. Also, the requirement by GFTAM for Tanzania to drop its proposal for a programme for orphans and children and instead undertake one on an antiretroviral treatment programme [46]. The failure to contain the spread of the recent Ebola outbreak in West Africa has been associated with the global health governance mechanisms implemented through GHIs that have predetermined focuses to the detriment of health promotion and health systems strengthening in countries [7]. The tide is changing, however. Lesotho’s approach to the implementation of the new strategy for HIV prevention through voluntary medical male circumcision stands out as a good example of how countries can challenge the traditional structures of global health politics controlled by experts and funders from high income countries [50]. To make an informed, local decision, Lesotho’s policy-makers consulted national statistics to determine if male circumcision was an effective approach to addressing the spread of HIV [50].

PEPFAR has been criticised for failing to link its objectives with those of the national strategic plans of the countries it supports [36, 40]. There is also evidence that it is global earmarks and donor conditionality that drive funding allocations regardless of countries’ diseases, health needs or priorities [1, 40]. A study conducted across Mozambique, Uganda and Zambia found that PEPFAR provided consistent and same level of funding allocations in the three countries regardless of their epidemiological and health systems’ differences [18]. According to Mckinsey and Company, BMGF 2005 [37], the countries with strong integrated health plans, established funding mechanisms with donor participation, and clearly defined roles of central and district governments interact the best with GHIs. This was also seen in an evaluation of MAP that attributed the failure of its approach to countries’ lack of national plans prioritising the components of their HIV/AIDS programme [51].

The multisectoral approach to HIV/AIDS is recognised for its success and cited as an example to follow for other disease conditions. However, how that approach is implemented has been the subject of extreme argument among health departments that feel disempowered or are bypassed by some of the GHIs such as MAP that favour that approach [51]. The failure to understand that the HIV response was multisectoral led many countries to ignore World Bank efforts to alleviate HIV/AIDS [51]. GHIs such as GFTAM also are faced with challenges for their insistence on planning processes that involve the country coordinating mechanism, a mutisectoral, private–public committee. There was lack of trust between government agencies and NGOs, and in some instances governments did not want to recognise the coordinating mechanism [18]. There were concerns that some of the country coordinating mechanisms lacked the capacity to function satisfactorily and were influenced by a few powerful members. The use of these mechanisms and the existence of several of them for HIV made it difficult to achieve alignment [36]. The presence of separate planning structures for the GHIs and the countries has led to duplication of effort [36]. Some GHIs have attempted to improve the harmonisation of their approaches with the country planning processes. This has been done through several strategies, as outlined in Table 2.

**Table 2:** Examples of GHI influence in planning and coordination in some African countries

Approaches	Examples
Medium- to long-term plans	In Angola GHIs support helped in the identification of appropriate measures for controlling the HIV/AIDS epidemic and for developing medium- to long-term plans.
Integration to national strategic objectives	In Rwanda GHI-supported activities have been integrated into the national strategic objectives, contributing to long-term sustainable interventions such as community health insurance schemes.
Influence on national and sub-national planning	GHIs have had positive effects on national and sub-national planning processes for HIV/AIDS in Zambia and Mozambique.

### Donor harmonisation and coordination

For aid to be effective there needs to be harmonisation of the processes between donors and other partners at the country level [52]. Harmonisation of donor policies and practices and their alignment with national policies have occurred at various levels across the countries. The negative effects of poor donor harmonisation were reported in the early years of the GHIs [18]. Despite the efforts of the countries to improve coordination and harmonisation of all donors, partners and GHIs, in the real sense GHIs are rarely part of these initiatives, as they tend to operate through separate systems and cycles or schedules [45]. This is further compounded by the lack of in country presence and as such, not able to participate in country dialogues in a consistent manner. To date little harmonisation has occurred of GFTAM processes and pre-existing planning and funding mechanisms such as SWAp and joint interagency committees [26, 53]. Evidence shows that GFATM’s requirement of separate reporting systems is associated with higher transaction costs [17, 22]. PEPFAR’s conditions, lack of transparency and unwillingness to involve other donors in its planning processes are cited as hindrances to harmonisation and collective donor action [18, 40].

The literature shows that over time improvements in harmonisation have occurred, with studies from 2004 to 2005 from across the countries indicating that GHIs are harmonising their approaches [49]. GFTAM’s agreement of 2004 to allow its funds to be channelled through Mozambique’s SWAp, the common fund, was seen as a pioneering example of how disease-specific programmes could learn and adapt [40]. A review of the Mozambican approach highlighted the fact that pooling of funds and participation in SWAp structures had given GFTAM a unique perspective on the Mozambican health sector, whilst enabling it to become a more harmonised and highly influential development partner [40]. Another example is the support of harmonisation in Nigeria and Namibia by GFTAM [18]. MAP projects through the World Bank have made several attempts to harmonise efforts in the countries, for example in Malawi, where they supported the country’s integrated service delivery approaches for pooling of resources and the national AIDS councils (NACs) [54]. Despite the improvement in the collaboration between PEPFAR and national systems in information sharing, there is still reluctance among GHIs to use national systems such as those of the Ministry of Finance or the reporting systems. PEPFAR has been reported as not being transparent with other GHIs working in the areas it deals with [40].

Monitoring and evaluation (M&E) of GHIs still is not merged with the national systems, which means that multiple M&E reports are being prepared at the national and district levels [11] with different requirements for the various programmes [37]. All the GHIs conduct their separate assessments, but often involve the same constrained health human resources available at the national and sub-national levels.

The literature shows contrasting perceptions of GFATM’s alignment with existing country M&E systems. In Cambodia, Uganda and Cameroon the use of GFATM project monitoring tools undermined national programmes and the Three Ones principle that requires countries use a single M&E system [14]. PEPFAR implementers collect large amounts of data that they do not generally share with government coordinating bodies or other donor agencies [18]. The World Bank, in its case, overburdens governments with extensive and complex procedural and reporting requirements for its MAP projects instead of using one strategic framework, one national authority and one M&E system [40].

The increasing funding for some specific health problems such as HIV/AIDS has forced many countries to make efforts for greater coordination of programmes and services at the national level. Several countries in Africa like Malawi, Zambia and Tanzania have benefited from the push by GFATM to institute mechanisms for coordination of actors at the national level. However, the incentives to ensure the functioning of the coordination efforts are weak and practice falls far short of the intent of the policy, especially at the sub-national level [40]. Further, involving all the relevant stakeholders, particularly NGOs, in coordination bodies is a challenge in many countries. The McKinsey study (2005) reported in Biesma et al. [18] found that Tanzania and the Democratic Republic of the Congo had at least four committees overseeing HIV/AIDS control, with little communication or commonalities among them.

Some positive effects of GHIs on coordination and planning have been reported. In Malawi for example, after the study of the policy project by the United States Agency for International Development in 2004 raised concern about the multiplicity of HIV/AIDS coordinating structures, the Malawi Partnership Forum was created in 2005 as the central coordination structure for development partners, overriding all other mechanisms [54].

### GHI contribution to stakeholder involvement

By actively involving NGOs and CSOs in their programmes, GHIs have changed the mind-set and perception that health delivery is the responsibility of governments [55]. Almost all GHIs tend to involve the private sector in their work, using several strategies and mechanisms. PEPFAR, for example, avoids the public sector in channelling its funds, choosing to use mainly international NGOs that fund CSOs [40, 56]. The Stop TB Partnership emphasises inclusive governance that incorporates the private sector, while GFATM’s country coordinating mechanisms have private sector representation on their committees. GHIs have been more effective than other financing mechanisms in diversifying stakeholder participation and involving NGOs and faith based organisations, enabling them to gain direct access to financial resources [45, 57]. Studies conducted in Malawi, Benin and Zambia showed that opportunities provided by GFATM strengthened public–private collaboration through allowing NGOs to establish umbrella organisations that helped to channel funds through principal recipients to sub-recipients [57]. There are still gaps, however, in the involvement of the private sector, and perceptions about how well GHIs are working with other stakeholders are contradictory [19]. There is evidence that some of the NGOs and CSOs do not have the capacity to implement GHI activities or absorb their funds [55]. Also, some indigenous NGOs have not been targeted nor reached with GHI funding, meaning that a few NGOs and CSOs dominate and benefit from GHI funding [15].

## Discussion

GHI commitments have been timely in providing the support necessary to handle diseases affecting LMICs [2, 41]. It is indisputable that without their support, coverage and access to disease-specific services such as those for HIV, tuberculosis and malaria would not have been possible for the LMICs [1, 41]. GHI support in LMICs has been varied in nature, involving financial support, technical assistance and HSS [17, 20, 24].

The positive outcomes of GHI support have been accompanied with negative effects that have generated controversy about its value [18, 19, 45]. GHIs have been criticised for their influence on national health priorities, decreased domestic spending on health, misalignment between their priorities and country health needs, distraction of government officials from their general responsibilities for health, creation of parallel systems, internal migration of health workers, increased burdening of HRH, lowered quality of services owing to pressure to meet targets, and weak accountability of the nongovernmental sector they fund [2, 20, 21, 24, 27, 56]. Despite being pro-poor, GHIs have not been able to directly address equity through poverty-reduction strategies, which has contributed to the growing inequalities in access to health services [12, 15, 22].

The African Region is continually striving to improve harmonisation and coordination of support from donors and partners [58]. The initiatives focusing on this include the Paris Declaration on Aid Effectiveness, the Accra Agenda for Action, International Health Partnership and HHA. All these have the common objective of ensuring that there is effective investment in low and middle income countries through financing of sustainable development initiatives. Such an objective can only be achieved by ensuring that aid goals are aligned with countries’ needs and priorities. It also requires that the countries have the upper hand in the decision and implementation of programmes, and that capacity is built and systems are strengthened for GHIs to work without affecting government functioning [59, 60].

### Study limitations

Rapid reviews are generally criticised for not being thorough in their searches. In order to mitigate this we conducted a thorough search using several sources of information including reference list so as not to miss any relevant study. Inherent in all literature reviews is the fact that, rigorous analysis is sometimes hampered by the content of existing studies. This was one of the main limitations of this study, since many of the recent studies had a similar study focus as previous ones. Despite this challenge, the review is an adequate reflection of the state of governance, priorities, harmonisation and alignment of GHIs in this setting.

## Conclusion

This rapid review of literature, which includes studies from GHIs’ early and recent times, reveals that little has changed in their approaches. GHIs still operate in a vertical manner, bypass countries’ systems, compete for the limited human resources, influence countries’ policies and favour unsustainable interventions [10, 13, 45]. The following recommendations will help to maximise the benefits and reduce the unintended challenges posed by the current GHI approaches:

- Future GHI support should be more comprehensive and less selective. It should include aspects for successful disease control such as provision of the necessary drugs through funding, donation or discounted pricing; funding for some operational costs; and technical assistance in line with identified systems gaps.
- There should be emphasis on strengthening of the wider systems that include social, community and health systems. GTZ’s BACKUP Initiative, which allocates specific funding for health systems support which is not disease specific [38], could serve as an example for this.
- GHIs should strive to improve country level governance by using existing structures such as SWAp or iHP+ mechanisms. This ideally should extend to the use of joint planning schedules, meetings and M&E systems in order to avoid duplication of effort and overburdening of the countries with parallel procedures.
- Modernisation is changing the disease profile in Africa. Africa is facing an alarming level of non-communicable diseases. GHI support should align with such changes with time. Also GHIs should evaluate their performance on epidemiologic effectiveness.
- GHIs should play an important role in promoting better governance in Africa through advocating for and stimulating generation and application of appropriate policies and approaches, and giving a special focus to transparency, accountability and performance.
- To improve the involvement of NGOs and CSOs, GHIs should support and promote standardisation of processes and accountability by CSOs at the country level and foster the empowerment of indigenous NGOs and CSOs.

## Abbreviations

CSOs, civil society organisations; DALY, disability-adjusted life year; GFATM, Global Fund to Fight AIDS, Tuberculosis and Malaria; GHIs, global health initiatives; GHP, global health partnership; HSS, health systems strengthening; MAP, World Bank’s Multi-country HIV/AIDS Program; NAC, national AIDS council; NGO, nongovernmental organisation; PEPFAR, US President’s Emergency Plan for AIDS Relief; WHO, World Health Organization.
